# Examining the Clinical Effectiveness of Non-Carbapenem β-Lactams for the Treatment of Extended-Spectrum β-Lactamase-Producing Enterobacteriaceae

**DOI:** 10.3390/antibiotics4040653

**Published:** 2015-12-15

**Authors:** Allison M. Gibble, Alan E. Gross, Angela M. Huang

**Affiliations:** 1 Department of Pharmacy, Froedtert & The Medical College of Wisconsin, 9200 W. Wisconsin Ave, Milwaukee, WI 53226, USA; E-Mail: allison.gibble@froedtert.com; 2 Department of Pharmacy Practice, University of Illinois at Chicago College of Pharmacy, 833 S. Wood St., 164 PHARM, Chicago, IL 60612, USA; E-Mail: aegross@uic.edu

**Keywords:** extended-spectrum beta-lactamases, piperacillin-tazobactam, cefepime, carbapenems

## Abstract

Treatment options for extended-spectrum β-lactamase-producing Enterobacteriaceae are limited. Piperacillin-tazobactam and cefepime represent potential alternative treatment options; however, large prospective studies are lacking. This review evaluates the current literature regarding use of piperacillin-tazobactam and cefepime for the treatment of extended-spectrum β-lactamase-producing Enterobacteriaceae. Antimicrobial stewardship programs can play a key role in guiding the best practices for the management of these challenging infections.

## 1. Introduction

Extended-spectrum β-lactamase-producing Enterobacteriaceae (ESBL-EB) are an increasing concern in both community- and healthcare-associated infections. Infections caused by these pathogens are associated with significant morbidity and mortality, and treatment options are limited [1]. Carbapenems (CBPs) have long been considered the treatment of choice for ESBL-EB. However, rising rates of resistance among Gram-negative bacteria necessitate the judicious use of CBPs, as even brief exposure to these antimicrobials can increase the risk of colonization or infection with a CBP-resistant organism [2,3]. Unfortunately, data evaluating the effectiveness of alternative agents, especially non-CBP β-lactams, is controversial.

β-Lactam/β-lactamase inhibitor combinations (BLBLIs), such as piperacillin-tazobactam (PT), are a potential alternative for the treatment of ESBL-EB. Several studies demonstrate *in vitro* activity of PT against ESBLs; however, clinical efficacy data are limited. Although ESBL-EB are generally inhibited by tazobactam, many of these organisms harbor additional resistance mechanisms such as AmpC β-lactamases that may limit the efficacy of PT [4]. To complicate matters, an inoculum effect may occur where PT MIC increases with bacterial inocula over 10 colony-forming units per milliliter; this is due to the high bacterial load and associated β-lactamase production overwhelming tazobactam. In time-kill studies, PT did not demonstrate equivalent bactericidal activity against ESBL-producing and non-ESBL-producing *Klebsiella pneumoniae*, despite having the same MIC [5]. In fact, two observational studies have reported treatment failures with PT in the treatment of ESBL-producing *K. pneumoniae* [6,7]. While PT may be less subject to inoculum effect than third- and fourth-generation cephalosporins, this phenomenon has been observed in strains of TEM-, SHV- and, less commonly, CTX-M-14 derived ESBLs and thus causes concern for its use in severe infections [8,9]. Cefepime, an oxyimino-cephalosporin and zwitterion, has enhanced activity against Enterobacteriaceae in part due to relatively low susceptibility to degradation by chromosomal and plasmid-mediated β-lactamases including some ESBLs. However, minimum inhibitory concentrations (MICs) for Gram-negative organisms harboring ESBLs may be increased and may often confer resistance [10]. In 2010 and 2014, the Clinical Laboratory Standards Institute (CLSI) lowered the susceptibility breakpoints for cefepime and introduced a new susceptible-dose-dependent category [11]. This change was made to capture Enterobacteriaceae unlikely to harbor ESBLs within the new susceptible range. Furthermore, based on animal models, it appears ƒT > MIC influences rapidity of bacterial killing more so than presence of ESBL [10,12]. Approximately 20% of Enterobacteriaceae may still remain susceptible to cefepime by current interpretive criteria despite the presence of ESBLs [13,14]. Even with the CLSI changes, clinicians remain skeptical of the utility of cefepime to treat susceptible ESBL-EB.

The purpose of this clinical review is to evaluate the available literature describing clinical outcomes of CBP-sparing β-lactam therapies for the management of infections caused by ESBL-EB.

## 2. Piperacillin-Tazobactam

Data examining PT for the treatment of ESBL-EB, specifically compared to CBPs, are limited to retrospective analyses (Table 1). Furthermore, studies vary significantly in treatment strategy, study method (examining empiric *vs*. definitive therapy), microbiological approaches, and outcome measures. Nonetheless, these studies provide valuable experience with the use of PT for these complicated infections.

**Table 1:** Studies evaluating the clinical outcomes of patients with extended-spectrum β-lactamase producing Enterobacteriaceae infections treated with non-carbapenem β-lactams.

Author, Year	Study Design, Location	Type of Infection	Pathogen(s)	Study Groups	Outcomes	Comments
**β-Lactam/β-Lactamase-Inhibitor Studies**						
Rodríguez-Baño, J., 2011 [15]	Post-hoc analysis of 6 prospective cohort studies, Spain	Bacteremia from any source	*E. coli*	ET: BLBLI (*n* = 72) CBP (*n* = 31) DT: BLBLI (*n* = 54) CBP (*n* = 120)	30-day mortality: ET: CBP 19.4% *vs.* BLBLI 9.7%, *p* > 0.2 DT: CBP 16.7 *vs*. BLBLI 9.3%, *p* > 0.2 No association between ET or DT with a BLBLI and increased mortality.	Most patients with low inoculum infections (urinary or biliary sources).
Kang, C.L., 2012 [16]	Retrospective observational cohort study, South Korea	Bacteremia from any source	*E. coli*, *K*. *pneumonia*, *K. oxytoca*	ET: BLBLI (*n* = 36) CBP (*n* = 78)	30-day mortality: CBP 26.9% *vs.* BLBLI 22.2%; *p* = 0.592. 3-day clinical response: CBP 81.4% *vs.* BLBLI 86.7%; *p* = 1.00. 7-day clinical response: CBP 64.7% *vs.* BLBLI 65%; *p* = 0.98. No association between empiric BLBLI and increased mortality.	Antimicrobial agent used for definitive therapy was not reported.
Tamma, P.D., 2013 [17]	Retrospective observational cohort study, United States	Bacteremia from any source	*E. coli*, *K. pneumoniae*, *K. oxytoca*, *P. mirabilis*	ET: BLBLI (*n* = 103) CBP (*n* = 110) DT: CBP (*n* = 213)	14-day mortality: CBP 8.0% *vs.* BLBLI 17.0%. Empiric BLBLI shown to be associated with increased 14-day mortality (HR, 1.92; 95% CI, 1.07 to 3.45; *p* = 0.03)	Variable dosing strategies were used for BLBLI.
Harris, P.N., 2015 [18]	Retrospective, observational cohort study, Singapore	Bacteremia from any source	*E. coli*, *K. pneumoniae*, *K. oxytoca*	DT: BLBLI (*n* = 24) CBP (*n* = 23)	30-day mortality: CBP 17.4% *vs.* BLBLI 8.3% (HR, 0.91; 95% CI, 0.13 to 6.28; *p* = 0.92); Length of stay: BLBLI 15 d *vs.* CBP 11 d (HR, 0.62; 95% CI, 0.27 to 1.42; *p* = 0.26)	The agent chosen for empiric therapy was not controlled by the study protocol.
Ofer-Friedman, H., 2015 [19]	Retrospective observational cohort study, United States and Israel	Bacteremia from non-urinary sources	*E. coli*, *K. pneumoniae*, *P. mirabilis*	ET and DT: BLBLI (*n* = 10) CBP (*n* = 69)	90-day mortality: CBP 48% *vs.* BLBLI 80% (OR, 4.5; 95% CI, 1.01 to 34; *p* = 0.05). 30-day mortality: CBP 34% *vs.* BLBLI 60% (OR 3.0; *p* = 0.1). BLBLI shown to be associated with increased 90-day mortality (OR, 7.9; 95% CI, 1.2 to 53; *p* = 0.03)	Breakpoints for pathogens are not reported.
**Cefepime Studies**						
Goethaert, K., 2005 [20]	Retrospective cohort study, Belgium	Pneumonia, 64%; bacteremia, 16%, and other	*E. aerogenes*	ET and DT: Cefepime (*n* = 21) CBP (*n* = 23)	Infection-related mortality: ET: CBP 26% *vs.* Cefepime 33%, *p* = 0.437	All patients received combination therapy with another agent. Cefepime 2 g q8h or equivalent dosing used.
Chopra, T., 2012 [1]	Retrospective cohort study, United States	Bacteremia from any source	*E. coli, K. pneumoniae*	ET: Cefepime (*n* = 43) CBP (*n* = 14) DT: Cefepime (*n* = 9) CBP (*n* = 33)	In-hospital mortality: ET: CBP 36% *vs.* Cefepime 40%, *p* = NS DT: CBP 36.4% *vs*. Cefepime 33.3%, *p* = NS No association between choice of therapy and mortality or length of stay in multivariate analysis.	Data from patients receiving either monotherapy with either cefepime or carbapenem shown. Cefepime dosing not described.
Lee, N.Y., 2013 [21]	Retrospective case-control study, Taiwan	Bacteremia from any source	*E. coli*, *K. pneumoniae*, *E. cloacae*	ET: Cefepime (*n* = 21) CBP (*n* = 91) DT: Cefepime (*n* = 17) CBP (*n* = 161)	30-day mortality: ET for those with initial appropriate therapy: CBP 17.9% *vs.* Cefepime 58%, *p* < 0.001 DT: CBP 16.8% *vs*. Cefepime 58.8%, *p* < 0.001 Propensity score-based matched analysis identified DT with cefepime as an independent predictor of 30-day mortality (adjusted OR, 6.8; 95% CI, 1.5–31.2)	Sepsis-related, 30-day, and crude mortality rates were greater as the cefepime MIC increased. Cefepime underdosed for patients infected with elevated MIC organisms; 1 g IV q8h or 2 g IV q12h used.

BLBLI, β-lactam/β-lactamase-inhibitor; CBP, carbapenem; DT, definitive therapy; ET, empiric therapy; MIC, minimum inhibitory concentration; NS, not significant.

One of the largest and most robust studies examining the use of BLBLIs *vs.* CBPs for the treatment of ESBL-EB was conducted by Rodríguez-Baño *et al*. [15]. This group performed a *post hoc* clinical outcomes analysis of 6 previously published cohorts in 192 patients from Spain receiving BLBLI (PT or parenteral amoxicillin/clavulanate) or CBPs for ESBL-producing *Escherichia coli* bloodstream infections (BSIs). The most frequently isolated ESBLs were CTX-M-14, followed by SHV-12 and CTX-M-15. The authors found similar 30-day mortality rates between those treated with a BLBLI *vs.* a CBP both in the empiric therapy cohort (9.7% *vs*. 19.4%, *p* = 0.1) and the definitive therapy cohort (9.3% *vs*. 16.7%, *p* = 0.1). Nosocomial acquisition of infection was significantly higher in patients empirically treated with CBPs (77% *vs*. 36%, *p* < 0.001) and more patients receiving carbapenems had severe sepsis or septic shock at presentation, suggesting that this cohort was more severely ill. In multivariate analysis and adjustment for a propensity score for receiving empirical CBP therapy, treatment group was not associated with mortality (*p* = 0.84) or length of stay. The authors concluded that definitive therapy with BLBLIs should be considered acceptable alternatives to CBPs for patients with BSIs due to ESBL-producing *E. coli* once susceptibility results are known. The results of this study are encouraging however generalizability of findings to high inoculum infections is difficult given most patients were bacteremic from urinary or biliary sources. This is an important limitation due to the potential for diminished activity of BLBLIs in high inoculum infections [8,9]. Furthermore, as this study was limited to *E. coli*, results may not be applicable to other Enterobacteriaceae (*Klebsiella* spp., *Proteus mirabilis*) that harbor different ESBLs. Because TEM- and SHV- ESBLs are more prevalent among non-*E. coli* Enterobacteriaceae, PT may be a less favorable option in high inoculum infections secondary to these organisms [22]. Interestingly, the authors observed a correlation between increasing PT MIC and mortality, with no deaths occurring in patients with a PT MIC of ≤2 mg/L. This may demonstrate suboptimal achievement of PK/PD targets as patients received non-extended infusion PT 4500 mg every 6 h. Use of extended-infusion BLBLIs may improve achievement of free-drug concentration time above the MIC (*f*T > MIC) targets in this setting although further studies are needed to confirm this. To date, this is the only study to evaluate both empiric and definitive PT and CBP therapy for ESBL-EB BSIs.

Kang *et al* reported a single-center, retrospective cohort study performed in South Korea analyzing empiric PT therapy compared to CBPs for the treatment of ESBL-producing *E. coli* and *K. pneumoniae* BSIs [16]. Of the 114 patients included, 36 received empiric PT therapy and 78 received empiric CBP therapy. No difference in 30-day mortality was found between the PT and CBP groups in univariate analysis (8/36 (22.2%) *vs.* 21/78 (26.9%, respectively), *p* = 0.592) and multivariate analysis showed no association with empiric PT therapy and increased mortality (OR, 0.55; 95% CI, 0.16–1.88; *p* = 0.343). After adjustment using propensity scoring for receiving empiric CBP therapy, there remained no increased risk for mortality in the PT group (OR, 0.63; 95% CI, 0.17–2.27). Furthermore, no significant difference in clinical response was found between the two groups at 3 days (*p* > 0.999) and 7 days (*p* = 0.983) after treatment initiation. The results of this study were presented as a “Letter to the Editor” in response to a previously published article. Because of this reporting method, many details of the study that are crucial to draw clinically applicable conclusions are missing, including sources of infection and definitions of outcomes. Additionally, because only 13 patients received definitive therapy with PT, the sample size was insufficient to perform an analysis examining impact of definitive therapy on clinical outcomes. While these results appear to support those found by Rodríguez-Baño *et al*, large gaps in information limit the ability to draw meaningful conclusions from this study.

In 2015, Harris *et al* performed an analysis of definitive therapy with BLBLIs *vs.* CBPs in ESBL-EB BSIs originating from any source [18]. This group conducted a single-center, retrospective, observational study of 91 patients in Singapore with BSIs caused by cefotaxime-resistant *E. coli* or *K. pneumoniae*. Patients were routinely deescalated from meropenem or imipenem to ertapenem or from PT to parenteral amoxicillin-clavulanate based on susceptibility results. The most common source of bacteremia was the urinary tract (47%). After adjustment for confounders, no difference in time to resolution of systemic inflammatory response syndrome (SIRS) was found between the treatment arms (HR, 0.91; 95% CI, 0.13 to 6.28; *p* = 0.97). No differences were found between treatment groups for multiple secondary endpoints including 30-day all-cause mortality, length of stay, incidence of *Clostridium difficile* infection, relapse of BSI, and isolation of a CBP-resistant isolate. Although the results of this study appear to support BLBLIs as an effective alternative to CBPs for the treatment of ESBL BSIs, there are many limitations to this study. Aside from small sample size, overall occurrence of all clinical outcomes was low in both cohorts, which may have impacted the ability to identify potential differences between the treatment groups. Finally, the retrospective design of the study contributes to a high likelihood of potential confounders. The tendency of prescribers to utilize carbapenem therapy in more severely ill patients may have contributed to overestimating the relative efficacy of BLBLIs.

In 2015, Ofer-Friedman *et al* published the only study analyzing the efficacy of definitive PT therapy as compared with CBPs for the treatment of EBSL-EB (*E. coli*, *K. pneumoniae* and *P. mirabilis*) BSIs originating from non-urinary sources [19]. This multi-center, international (Detroit, Michigan and Israel), retrospective cohort analysis included 69 patients who received CBPs and 10 patients who received PT. Treatment with PT was associated with increased 90-day mortality (80 *vs*. 48%; OR, 7.9; *p* = 0.03), longer ICU and hospital length of stay and deterioration in functional status; however, a statistically significant difference between the two cohorts was reported only for 90-day mortality. In multivariate analysis, after controlling for risk of being given PT, PT administration remained an independent predictor of mortality. However, results of the multivariate analysis are difficult to interpret, as variables included in the regression model were not well-defined. An underlying goal of this study was to further explore the conclusions made by the Rodríguez-Baño analysis but instead focus on non-urinary source BSIs. Although this was a small study with notable limitations in study design, the results highlight that source of infection may impact outcome when determining treatment choice in patients with ESBL-EB [19].

The largest retrospective review examining the impact of empiric therapy of ESBL-EB was conducted by Tamma *et al* in 2015 [17]. This single-center study conducted in Baltimore, Maryland included 213 patients who received either PT or a CBP as empiric therapy followed by definitive CBP therapy as treatment for BSIs caused by an ESBL-EB. In multivariate analysis, empiric PT was associated with a 1.92 times higher risk of death at day 14 compared to CBP therapy (95% CI, 1.07–3.45). This analysis provides insight into the impact of empiric therapy on ESBL-EB. Interestingly, results of this study contrast with those found by Rodríguez-Baño *et al*, who report empiric use of CBP to be equivalent to PT. The discrepancy may be related to differences in source of infection, baseline severity of illness, implicated organisms, and regional epidemiology [15]. The study by Tamma *et al* included a variety of pathogens in addition to *E. coli*, such as *K. pneumoniae*, *K. oxytoca*, and *P. mirabilis* [17]. As stated previously, non-*E. coli* Enterobacteriaceae frequently harbor several subtypes of ESBLs which may have shifted favor to empiric CBP therapy [23]. This study also included patients with high inoculum infections originating from sources such as the lower respiratory tract, which may affect the efficacy of PT. Lastly, epidemiological differences in pathogens isolated from Spain and the United States limit the ability to generalize results to different regions and may in part account for discrepancies seen in clinical outcome. Of note, variable dosing regimens for PT were used throughout the study and extended-infusion administration was not used, which has been shown to decrease mortality especially in patients at high risk of death [24]. Variations in dosing strategies may have impacted the ability of these patients to achieve adequate probability of pharmacodynamic target attainment and thus ultimate clinical outcomes.

Despite the limitations of the aforementioned studies and heterogeneity of the designs, it is apparent that there may be an association with ESBL-EB BSIs from high inoculum sources, specifically in those with a complex genetic profile, and improved outcome with CBP therapy. The clinical challenge that remains is how to identify these patients and target therapy prior to organism identification and susceptibility results.

Of note, a randomized controlled trial comparing meropenem *vs.* piperacillin-tazobactam for definitive treatment of blood stream infections due to ceftriaxone non-susceptible *E. coli* and *Klebsiella* spp. is currently recruiting participants. The primary outcome to be analyzed is 30-day mortality in addition to multiple secondary outcomes including time to clinical and microbiologic resolution of infection, clinical and microbiologic success, microbiologic resolution of infection, microbiologic relapse, and superinfection with a carbapenem- or piperacillin-tazobactam-resistant organism or *C. difficile* [25].

## 3. Cefepime

In the 2014 CLSI revision, the interpretive criteria for Enterobacteriaceae and cefepime were updated to reflect the observed poor clinical outcomes in patients infected with pathogens with a cefepime MIC ≥8 mcg/mL when cefepime doses of 1–2 g IV every 12 h are used [10,11,26]. The revised breakpoints reflect the importance of optimizing dosing in organisms with elevated MICs. With the revised breakpoints, Enterobacteriaceae with a cefepime MIC ≤2 mcg/mL are considered susceptible, 4–8 mcg/mL is considered susceptible-dose dependent (necessitating cefepime 2 g IV every 8 h), and ≥16 mcg/mL is resistant. Despite this change, many clinicians remain skeptical of the utility of cefepime for the treatment of ESBL-EB as this may result in treatment failures [27]. Because of the limited available treatment options for ESBL-producing Enterobacteriaceae infections and the need to preserve CBPs as last line agents, the effectiveness of cefepime in the treatment of ESBL-EB has been the primary objective of numerous studies [1,20,21,28,29,30]. Similar to PT, there are limited data evaluating the efficacy of cefepime *vs.* CBPs in the treatment of ESBL-EB infections (Table 1).

Lee *et al* reported the outcomes of a retrospective cohort of patients treated with cefepime or a CBP for monomicrobial ESBL-EB BSIs in two hospitals in Taiwan [21]. Pneumonia and indwelling catheters were the major sources of infection and *E. coli*, *K. pneumonia,* and *Enterobacter cloacae* were the included causative pathogens. Patients receiving cefepime (*N* = 17) as definitive therapy for organisms with an MIC ≤8 mcg/mL were compared to 161 patients treated with a CBP; in multivariate analysis, definitive cefepime therapy was an independent predictor of 30-day mortality (OR, 9.9; 95% CI, 2.8–31.9). And, a 1:1 propensity score-based matched analysis including patients treated with cefepime or CBPs as definitive therapy also revealed cefepime as an independent predictor of 30-day mortality (adjusted OR, 6.8; 95% CI, 1.5–31.2). An analysis of mortality rates stratified by cefepime MIC in patients receiving cefepime was also done. In the 33 patients treated with cefepime either as empiric or definitive therapy, sepsis-related, 30-day, and crude mortality rates were greater as the cefepime MIC increased; sepsis-related mortality in patients infected with organisms with MICs of ≤1 mcg/mL, 2–8 mcg/mL, and ≥16 mcg/mL were 0, 50%, and 71.4%, respectively (*p* = 0.006). This study suggests that cefepime definitive therapy may be less effective than CBPs for ESBL-SB BSIs. However, this study was conducted prior to the revision of CLSI breakpoints in 2014 and thus, patients infected with organisms with MICs of 2–8 mcg/mL received dosing regimens of either 1 g IV every 8 h or 2 g IV every 12 h and not the optimal dose of 2 g IV every 8 h. Suboptimal dosing limits conclusions from this study about the ineffectiveness of cefepime in this MIC range [31,32]. While cefepime may be a reasonable option for infections with adequate source control, efficacy in higher risk BSIs is yet to be elucidated.

Chopra *et al* compared the outcomes of cefepime *vs.* CBPs in a retrospective cohort of 145 patients with ESBL-producing *E. coli* or *K. pneumoniae* bacteremia in Detroit, Michigan [1]. Multivariate analysis did not reveal any association with mortality in patients that received cefepime monotherapy empirically (OR, 1.66; 95% CI, 0.71–3.87) or as definitive therapy (OR, 0.8; 95% CI, 0.34–2.29). Source of bacteremia was not well described, however patients with line-related BSIs were compared to all other sources and no association with mortality was found. In patients who received empiric cefepime monotherapy, no association between increasing cefepime MIC and mortality was found. Finally, there were no significant associations between type of empiric or definitive therapy and hospital readmission or length of hospitalization. These findings are in contrast to the study by Lee *et al* [21]; although no differences in outcomes were found when stratified by cefepime MIC or empiric treatment, these analyses were likely underpowered given only 43 and 9 patients received cefepime monotherapy empirically or as definitive therapy, respectively. Finally, cefepime dosing was not described in this study further limiting potential conclusions.

Goethaert *et al* evaluated outcomes of high-dose cefepime (2 g IV every 8 h) *vs.* CBP therapy for the treatment of ESBL-producing *Enterobacter aerogenes* infections in 43 intensive care unit patients in Belgium [20]. Pneumonia was the most common infection occurring in 64% (28/44) of episodes. In patients receiving cefepime or a CBP, there was no difference in clinical failure (38% *vs*. 30%, *p* = 0.592) or infection-related death (33% *vs*. 26%, *p* = 0.437). The major limitation of this study is the use of empiric combination therapy for all patients with amikacin or ciprofloxacin; this limits any conclusion about the relative treatment effect between CBP and cefepime. Furthermore, this study only included patients with *E. aerogenes* infections.

A small retrospective case-control study of patients receiving cefepime for non-urine *Klebsiella* spp. and *E. coli* infections was conducted by Kotapati *et al* in a single-center in the United States [28]. Ten patients with ESBL-positive isolates were matched to 20 patients with ESBL-negative isolates based on site of infection, ICU stay, pathogen and date of hospitalization. Pneumonia was the primary infection in 83% of the total cohort and all patients received cefepime dosed either as 1 g IV every 12 or 24 h. The clinical success rate was greater in the non-ESBL *vs.* ESBL group (87% *vs*. 40%, respectively; *p* = 0.002). Unfortunately, susceptibilities for cefepime were not reported in the non-ESBL group and 40% of the ESBL group had isolates resistant to cefepime (MIC ≥ 16 mcg/mL). Cefepime-resistant isolates were found in 4/6 of the patients who experienced clinical failures in the ESBL group. The other two failures occurred in patients infected with organisms with an MIC of 4 mcg/mL. All-cause and infection-related mortality was not different between groups however this analysis was underpowered. Although this study did not specifically evaluate use of cefepime as compared to CBPs, results show that treatment failure with cefepime is common in cefepime-resistant ESBL-EB. This finding is consistent with previous studies that have found increased mortality with delayed time to effective therapy for BSIs caused by Gram-negative bacteria [33].

Additional studies have assessed outcomes in patients treated with cefepime for ESBL-producing Enterobacteriaceae as a primary objective or as a study subgroup; but no conclusions can be drawn from these due to small populations studied in addition to other limitations [29,30,34,35]. As noted, the largest and most well conducted study by Lee *et al* suggested there should be significant concern for using cefepime for ESBL-producing Enterobacteriaceae given cefepime was an independent predictor of crude 30-day mortality compared to CBPs [21]. However, an unanswered question is whether cefepime is as effective as CBPs for ESBL-EB when the cefepime MIC is 2–8 mcg/mL and optimal dosing is used (2 g IV every 8 h) [31]. *In silico* pharmacodynamic simulations suggest an adequate probability of pharmacodynamic target attainment in this MIC range when 2 g IV every 8 h is used [36]. Additionally, in animal models, *f*T > MIC rather than ESBL-production has been associated with rapidity of killing [12].

Collectively, these limited retrospective studies with only small numbers of patients is likely a reflection of the understandable lack of confidence clinicians have in using cefepime for ESBL-producing organisms. No adequate clinical outcomes data exists to routinely recommend cefepime for the treatment of ESBL-EB infections. Although *in vitro* and *in vivo* data are encouraging and support the current breakpoints, when faced with a severely ill patient with a corresponding high risk of mortality infected with an EBSL-EB, it would be difficult and arguably inappropriate to recommend the use of cefepime instead of a CBP. However, in clinically stable patients with non-severe infections (e.g., cystitis) and source control, cefepime may be considered for ESBL-EB when susceptible*.* Although there is a clear need for stewardship of broad-spectrum antibiotics, if there is a risk that cefepime is in fact inferior to CBPs in these scenarios, then CBPs should be used.

## 4. Discussion

While CBPs are currently the standard of care for ESBL-EB infections, rising rates of Gram-negative resistance are highlighting the need for the judicious use of these agents. Cefepime and PT are potential alternatives to CBPs for the treatment of ESBL-EB, however the current data are limited to retrospective observational studies, most with small sample sizes and heterogeneous populations. Thus, available data investigating cefepime or PT therapy *vs.* CBPs for the treatment of ESBL-EB should be interpreted with caution. Significant differences in study methodology exist amongst studies, and therefore it is important to consider each study individually based on pathogens studied, type of infection, antibiotic dosing, use of concomitant antibiotics, and stratification of outcomes by empiric or definitive therapy. While all the PT studies examined patients with bacteremia, source of infection differed significantly with some studies including only high inoculum infections, whereas others included BSIs from any source. While the latter situations make results more generalizable, it becomes difficult to discern differences that may exist within certain risk groups. For cefepime, the data extends to non-bacteremia studies and these infections are often difficult to characterize retrospectively. Furthermore, cefepime dosing was not optimized in most studies. Differences in studied microorganisms also make interpretation of results problematic. Variation in ESBL subtype and expression of multiple β-lactamases are seen amongst different species of Enterobacteriaceae which may impact efficacy of PT or cefepime. Lastly, it is important to note regional differences in resistance patterns that may limit applicability of these results outside of the area in which the studies was conducted. For example, clinicians in regions with high rates of CMY-producing *E. coli* (which are commonly not inhibited by tazobactam) should use BLBLIs with caution, despite any positive results of the included studies [37].

Recently, the FDA approved two new broad spectrum BLBLIs, ceftolozane-tazobactam and ceftazidime-avibactam, for the treatment complicated urinary tract infections and complicated intra-abdominal infections. *In vitro*, both agents have activity against some ESBL-EB [38,39,40,41]. Similar to PT, the addition of tazobactam to ceftolozane inhibits ESBL production by binding to the active site and preventing BL hydrolysis [40]. Currently, there are no adequate clinical data comparing ceftolozane-tazobactam to CBPs for the treatment of ESBL-EB however it may be subject to the same concerns as PT in this setting. Avibactam is a non-β-lactam (diazabicyclooctane) β-lactamase inhibitor that has activity against Ambler class A and D ESBL-EB and has enhanced activity against AmpC and *Klebsiella pneumoniae* carbapenemase (KPC)-producing Enterobacteriaceae. Avibactam inhibits β-lactamase activity by forming covalent bonds [41]. Similar to ceftolozane-tazobactam, no adequate clinical studies have examined cefazidime-avibactam for the treatment of ESBL-EB. While these two agents represent potential alternative treatment options for ESBL-EB, more studies are required to fully elucidate efficacy compared to CBPs. Furthermore, because ceftazidime-avibactam is the only available β-lactam with activity against KPC-producing Enterobacteriaceae, its use needs to be reserved for that indication and should not be used as a means to spare carbapenem usage.

Overall, data supporting the use of PT or cefepime for the treatment of ESBL-EB infections are limited. Based on available literature, it appears that patients with high inoculum ESBL-EB infections may have worse outcomes when treated with PT and thus warrant CBP therapy. Conversely, patients with low inoculum ESBL-EB infections with a controllable source and who demonstrate *in vitro* PT susceptibility may be appropriately treated with PT for definitive therapy on a case-by-case basis. Doses should be optimized and extended-infusion should be used when possible to maximize the probability of pharmacodynamic target attainment [42]. Choice between empiric therapy with PT or a CBP remains challenging as early identification of patients at high risk for ESBL-EB infections is difficult. For cefepime, despite some data to support the current CLSI breakpoints, adequate clinical studies are lacking and thus cefepime cannot be recommended routinely for patients with ESBL-EB infections. In the context of ESBL-EB, cefepime may be considered as a second-line agent for non-severe infections where the isolate is susceptible, the source of infection can be controlled, and optimal dosing is used.

Due to increasing rates of antimicrobial resistance, optimizing treatment of ESBL-EB and other infections with complex resistance patterns should be a focus of antimicrobial stewardship programs (ASPs). Appropriate treatment depends on multiple factors including type and severity of infection as well as patient specific factors, and thus a thorough understanding of available literature is important. ASPs can improve patient outcomes by ensuring the optimal treatment of these infections.
